# Developing and centralising a nurse‐led local anaesthetic transperineal biopsy service during COVID

**DOI:** 10.1002/bco2.251

**Published:** 2023-05-31

**Authors:** David Adam Winters, Ashley Mehmi, Amar Odedra, Lydia Wilson, Joey Ancheta, Sally Buttleman, Paula Allchorne, Prabhakar Rajan, Shahid Khan, James S. A. Green

**Affiliations:** ^1^ Department of Urology Whipps Cross University Hospital NHS Trust London UK; ^2^ Department of Urology Newham University Hospital London UK; ^3^ Department of Urology St Bartholemew's Hospital London UK

**Keywords:** advanced nurse practitioner, biopsy, COVID‐19, prostate cancer, quality improvement, transperineal, transrectal

## Abstract

**Introduction:**

Transperineal (TP) biopsy has recently replaced the transrectal ultrasound (TRUS) approach as the ideal method of biopsy in the United Kingdom with growing trends to adopt. To minimise transmission of COVID‐19 during the first wave of the pandemic, the British Association of Urological Surgeons Section of Oncology issued guidelines reducing general anaesthesia (GA) procedures and initiate COVID‐secure ‘green’ site diagnostics. As a result of these guidelines and reduction in clinical diagnostics trust‐wide, we ceased all TRUS diagnostics and implemented a centralised, nurse‐led LA TP biopsy service.

**Materials and methods:**

A waiting list was developed for those awaiting prostate cancer diagnostics across the network. A COVID‐secure ‘green’ site was quickly identified with TP biopsies starting soon after. Quality improvement methodology was utilised and a run chart was used to show if changes were sustainable.

**Results:**

Successful implementation and centralisation of a TP biopsy service occurred with TRUS guided biopsies ceasing across all sites on 12 May 2020. The procedures were carried out by urology advanced nurse practitioners under local anaesthesia with a select few occurring under GA. Centralising the service in a COVID‐secure manner freed up dedicated theatre sessions and personal leading to increased efficiency elsewhere. The service was robust and was maintained upon lifting of COVID restrictions.

**Conclusions:**

A centralised, nurse led LA TP biopsy service in a procedural unit was implemented successfully. The service has remained resilient upon lifting of restrictions and return to business as usual. This led to improved performance across trust by freeing up valuable resources and staff to undertake more duties. The service remains highly valued trust‐wide.

## 1 INTRODUCTION

Transperineal (TP) template biopsy and transrectal ultrasound (TRUS)‐guided biopsy of the prostate are important tools in the histopathological diagnosis of prostate cancer in the United Kingdom.^1^ Recent data from Hospital Episode Statistics shows that over the last decade, there is a growing trend in selecting TP over the TRUS approach in cancer diagnostics.^1^, ^2^ When utilised with mpMRI, it can provide targeted biopsies with 93% sensitivity in diagnosis of Gleason 4 + 3 prostate adenocarcinoma and 87% sensitivity in the diagnosis of Gleason 3 + 4 prostate adenocarcinoma.^3^ Furthermore, there are other significant advantages of TP biopsy such as a lower risk of sepsis when compared with the transrectal approach and higher detection rates of cancer in the anterior prostate.^4^, ^5^ TP biopsy can be done with under general anaesthetic; however, more skilled practitioners opt for local anaesthesia. Despite these benefits, biopsy via the transrectal approach remains the most common type of biopsy in the United Kingdom.^6^ This may be due to a variety of reasons such as the cost of equipment, training requirements and increase in procedure time.

During the unprecedented COVID‐19 pandemic the British Association of Urological Surgeons (BAUS) section of oncology released specific guidelines in the management of suspected prostate cancer patients in March 2020.^7^ These recommendations were implemented to reduce the transmission of COVID‐19 amongst high‐risk groups and made proposals such as minimising outpatient attendance, minimising risks of sepsis and postponing general anaesthetic procedures.^7^ Subsequently, diagnostics were postponed at a number of trusts in accordance with these guidelines until appropriate alternative measures could be arranged.

The move from a transrectal to a TP biopsy approach was first laid out by Rick Popert from Guys a St Thomas' NHS Trust in 2017.^8^, ^9^ Bart's Health NHS Trust is one of the largest trusts in England accounting for 1.5% of all hospital activity.^10^ The Urology Department covers five separate hospitals, and in this article, we look at the expedited implementation of a trust wide Transperineal Biopsy Service in lieu of TRUS guided biopsy service, as per the BAUS guidance, to ensure high standards of care can be maintained despite the unprecedented COVID‐19 pandemic. Typically, at Barts Health NHS Trust, each hospital's urology department would carry out TRUS biopsy at their own site, with TP biopsy being utilised as a specialised test under general anaesthetic performed by a urology consultant at the Royal London Hospital. The indication for GA TP biopsy was often for re‐biopsy, mainly of active surveillance patients, patient preference or previous negative biopsies with a high index of suspicion for prostate cancer. Biopsies would occur on a weekly basis with six TP biopsies on average being performed over two sessions (8:00 am–12:30 pm and 1:30 pm–5:00 pm respectively).

We aim to see if the implementation of a TP biopsy service performed under local anaesthetic, in a day‐case procedure unit by a urology advanced nurse practitioner first could be implemented as a single bundle rather than in a step wise fashion while being safe and whether it can reduce waiting list pressures that were exacerbated by the pandemic, while reducing pressures on operating theatres and freeing up specialist theatre staff to undertake other GA operations. We aimed to look at data in real time to ensure that changes could be made so that this process can be both maintained after COVID restrictions are lifted and could be replicated amongst other trusts.

## 2 METHODS

TP biopsies had been carried out at a tertiary centre at the Royal London Hospital (RLH), part of Barts NHS Hospitals Trust since 2008. Referrals from across the trust were made to the RLH in the event of previous negative TRUS biopsy, and these were carried out under general anaesthetic.

### 2.1 Training advanced nurse practitioners

As part of Barts Health NHS trust's strategy to decrease the reliance on TRUS biopsy in August 2018, equipment for TP biopsies was moved to Whipps Cross University Hospital as part of the centralisation process and biopsies commenced under general anaesthetic in theatres in August 2018. This was done with a view to eventually phasing out TRUS biopsy as a first‐line diagnostic test, working closely with Guys a St Thomas' NHS Foundation trust^8^ to learn from their success. Barts Health Urology Advanced Nurse Practitioners (ANP's) based at Whipps Cross University Hospital commenced training in February 2019 at Guys and St Thomas' hospital. ANP (J. A.) was signed off on this procedural skill in August 2019, with nurse‐led TP biopsies under GA starting in January 2020 at Whipps Cross University Hospital, with Local anaesthetic procedures starting soon after. Nurses were trained using the PrecisionPoint Transperineal Access System developed by Perineologic, Cumberland, MD, USA.^11^ This method was chosen due to its ease of use and being well documented in peer‐reviewed literature as a safe, tolerable and effective method for TP biopsy.^12^, ^13^, ^14^

### 2.2 COVID‐19 lockdown and developing a centralised service

In March 23, the United Kingdom went into a national lockdown due to the COVID‐19 pandemic. Subsequent BAUS guidance was released to minimise the risk of spread, and prostate biopsies were put on hold. In March 2020, the decision was made to cease routine TRUS biopsies. GA TP biopsies performed in theatres by a urology consultant were also ceased at this point, and it was planned to expedite a centralised TP biopsy service as the first‐line standard in Barts Health NHS Trust at Newham University Hospital. This was carried out by trust‐wide consultant urologists and a lead urology specialty nurse with experience in project management and implementation of complex projects (PA) using quality improvement (QI) and implementation of science framework. Weekly meetings were held to ensure smooth transition and allowed for fast‐paced problem solving that may have arisen because of the pandemic.

A virtual waiting list was developed with the most urgent cases being identified for biopsy once service resumed. The patients on the waiting list near the time of lockdown were risk‐stratified as per the BAUS section of Oncology Covid‐19 strategy.^7^ Those with a PSA > 20 and considered high risk for metastases were initiated on anti‐androgen therapy to ensure their PSA remained stable until a safe date for TP biopsy could be arranged, mindful that this could affect histological grading of any tumours discovered.

### 2.3 Starting the service

The biopsies were to take place in a COVID‐secure manner, with all members adhering to Barts Health NHS Trust PPE guidance, with members of staff wearing full PPE; gloves, aprons and visors and patients having a negative COVID swab 3 days prior to the procedure as well as self‐isolating.^15^ Biopsies were undertaken in a stand‐alone COVID‐free site, called the gateway centre. The gateway centre is an orthopaedic day‐case theatre complex at Newham University Hospital and was quickly identified as a location in which Barts Health Urology could set up a COVID secure (‘green site’) as all clinical activities ceased early on during the pandemic. TP biopsies on the ‘gateway pathway’ started at the Newham University Hospital on 12 May 2020.

There were 56 patients waiting for a TP biopsy prior to the services start date consisting of 13 patients under routine active surveillance for previously diagnosed prostate cancer, 14 patients who had delays to an earlier biopsy, 26 patients of a 2‐week wait suspected cancer pathway and 3 patients who cancelled the procedure altogether. The waiting list consisted of patients being referred as early as the 8th of October 2019, and the last case we observed was referred on the 5th of November 2020. Once an efficient service was built with standard operating procedures in place, the TP biopsy service was relocated in January 2021 within the trust to a purpose built ‘green’ urology diagnostic and treatment centre at Whipps Cross University Hospital to streamline the service further. A small number of patients meeting certain criteria such as being unable to tolerate a GA procedure or those requiring repeat biopsies were still offered a GA procedure by a consultant urologist albeit at a significantly lower number.

**FIGURE 1:**
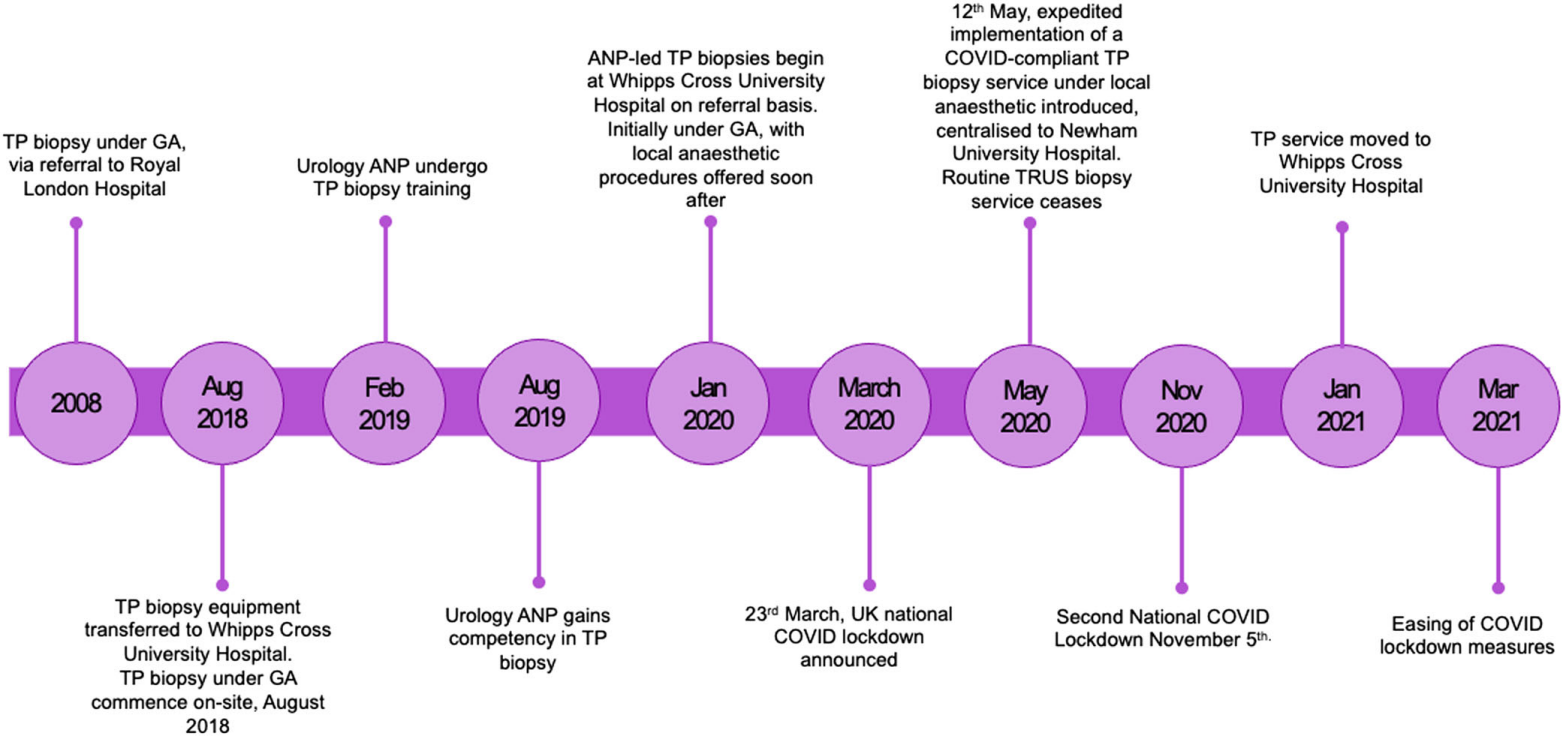
Timeline of service development.

The time between referral date and procedure completion was calculated using an Excel Spreadsheet, and waiting times were monitored in real time using a run chart to monitor service efficiency in real time so that changes could be made if required. Finally, quantitative analysis was used to calculate to see if there was a statistically significant reduction in the waiting times for prostate biopsy in roughly six monthly timeframes; pre‐lockdown (26 September 2019–23 March 2020), during strict lockdown measures (12 May 2020–5 November 2020) and upon easing of lockdown (1 June 2021–29 November 2021) (Figure 1).

To ensure no cases were missing and in order to review the gradual phase out of the TRUS service, a request was made to the Business Intelligence Unit (BIU) at Barts Health NHS Trust for all activity both TRUS and TP biopsies across all sites from January 2018 to November 2021. A search was carried out using the procedural codes a list of all procedures was sent to us. Each case was then validated by two doctors with experience in urology (D. W., A. O.) for quality control and to ensure each case was coded correctly.

## 3 RESULTS

The age range of patients undergoing biopsy where available was 40–85 years. Biopsies were requested for patients with mpMRI scans showing PIRADS 3 lesions or greater or those whose PSA density did not correlate with mpMRI prostate volumes.

### 3.1 Comparison of pre‐lockdown (26 September 2019–26 March 2020) to Lockdown (12 May 2020 to 05 November 2020)

A Shapiro–Wilk test showed that the data were not normally distributed when looking at pre‐ and post‐lockdown groups as a whole subjected to extreme outliers. We subsequently ran a Mann–Whitney *U* test. The results showed that there was a significant difference between the average waiting time pre‐ and post‐lockdown (*U* = 55.5, *p* ≤ 0.001). The mean waiting time for those in pre‐lockdown was 145 days whereas in post‐lockdown was 23 days. These results are illustrated in Figure 3.

### 3.2 Comparison of lockdown (12 May2020 to 05 November 2020) to post‐lockdown (01 June 2021 to 29 November 2021)

A Schapiro–Wilk test showed that this comparative data were not normally distributed, and we subsequently ran a Mann–Whitney *U* test. The results showed that the average waiting time for a TP biopsy during lockdown restrictions was 23 days (*n* = 144) and post‐lockdown and subsequent increase in clinical activity 30 days (*n* = 271). This difference was not statistically significant (*U* = 18461.5 *p* = 0.36812).

## 4 DISCUSSION

**FIGURE 2:**
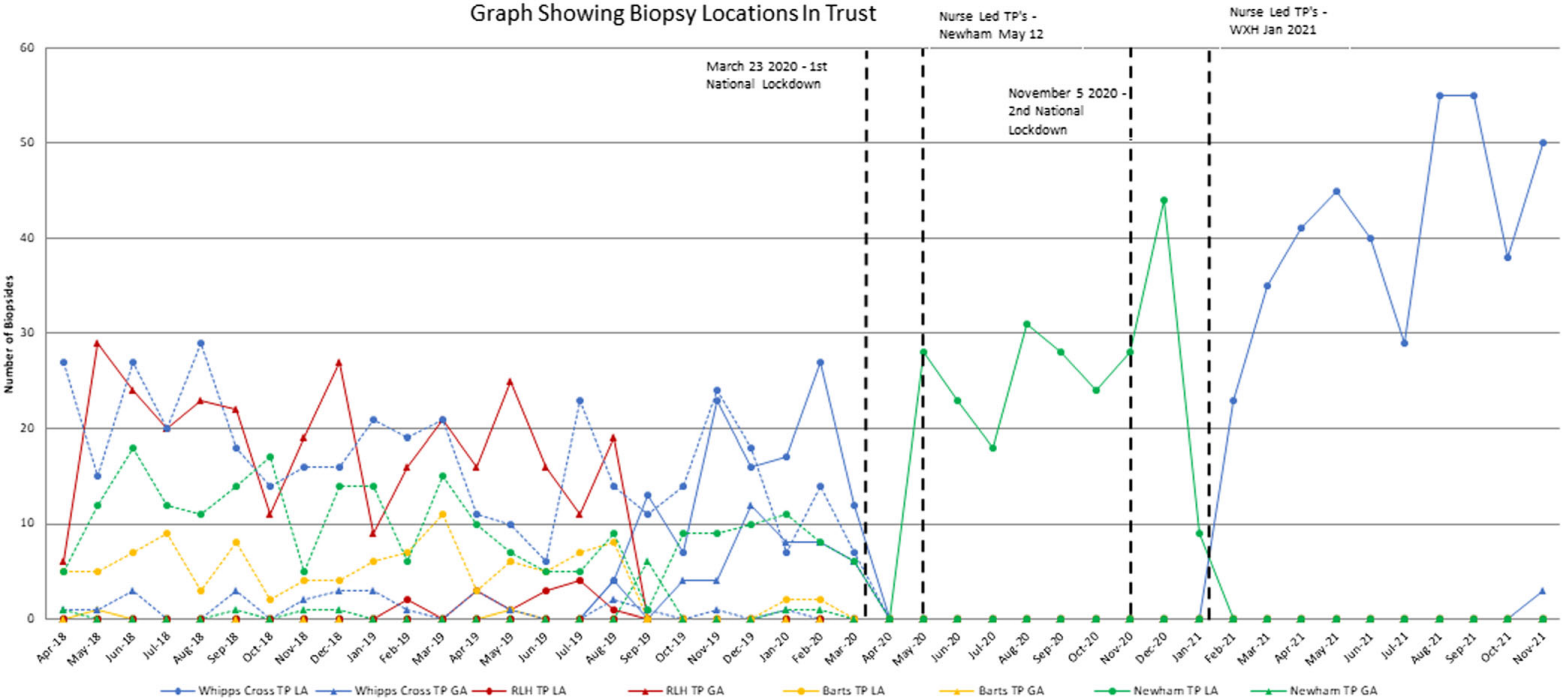
Graph showing all biopsy locations in the trust pre‐ and post‐lockdown.

When looking at Figure 2, it is clear to see the potential issues that may have arisen from a cross‐site multiprocedural prostate biopsy service. Figure 2 clearly demonstrates the complexity of managing prostate biopsy waiting lists spread over four sites with two biopsy techniques and demonstrates eight waiting lists managed by different teams administering theatres and outpatients. Furthermore, this type of service would require increased resources to ensure all patients on a suspected prostate cancer pathway are tracked and referred to the multi‐disciplinary team meeting. Streamlining of services can be viewed if Figure 2 can be simplified further if we look at TRUS and TP biopsies separately. As one can see multisite TRUS biopsies ceasing entirely after lockdown and TP biopsies being centralised to Newham and then Whipps Cross Hospitals respectively without any breaks in the service delivered. Figure 2 data show that up until January 2021, 100% of all TP biopsies were nurse led, under LA at a day‐case unit on a single site achieving our primary objective. Additionally, it shows that we have been able to successfully embed this service at Whipps Cross and has been able to continue when transitioning to business as usual (BAU).

**FIGURE 3:**
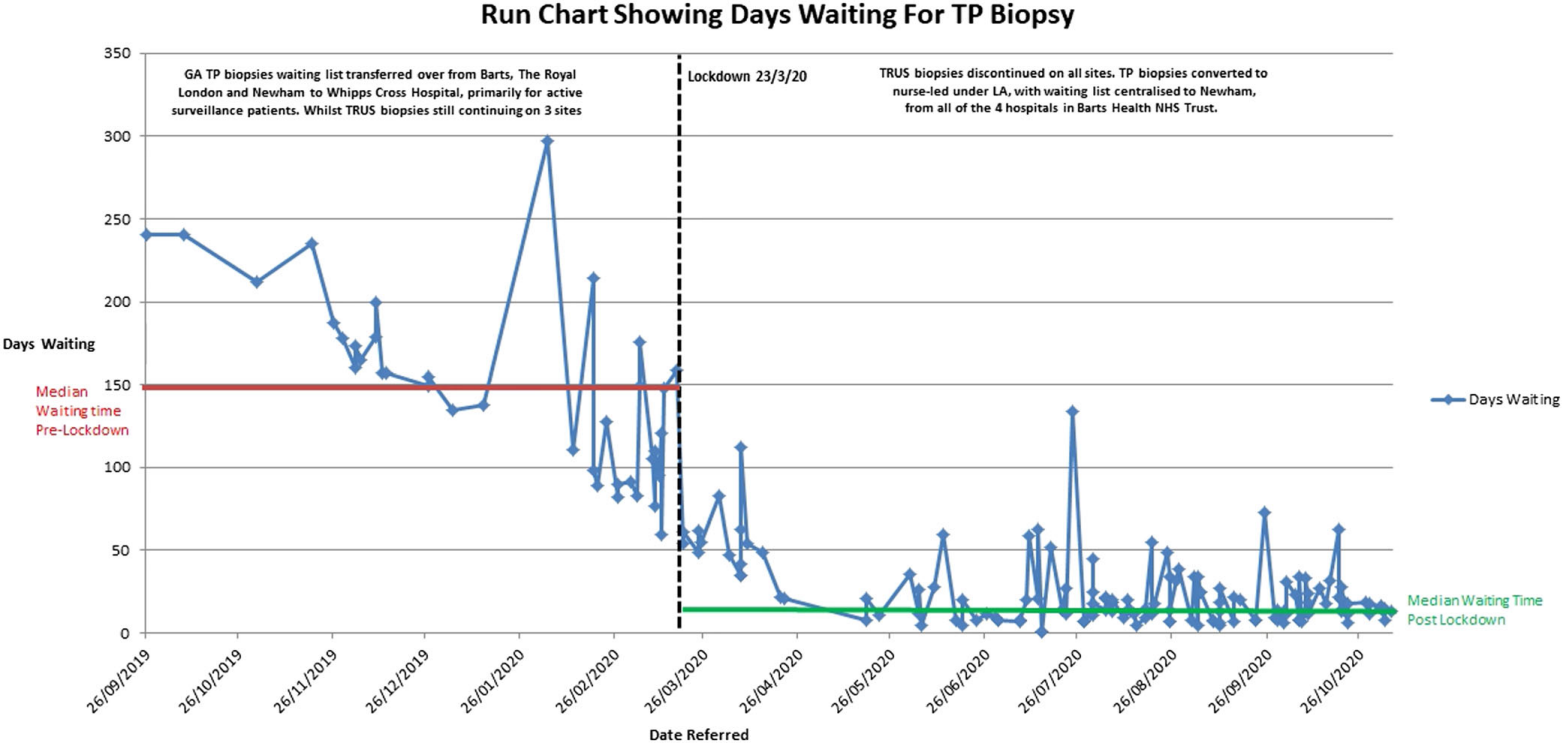
Run chart showing days waiting for transperineal biopsy pre‐ and post‐lockdown.

The positive impact on service waiting times is clearly visualised in the Figure 3 run chart. This chart shows the median wait time for TP biopsy specifically pre and post lockdown. It can be inferred that upon centralising the service to a single site, less potential issues may have arisen in terms of administration errors with further added benefit of streamlining the MDT as set out in the guidance by NHS England and GIRFT.^16^, ^17^ The reduction in median wait time with only small fluctuations from the median shows the service was run in a stable manner with relatively little intervention required.

Run charts are a useful tool in quality improvement as we are clearly able to visualise the impact before and after the implementation of a new service, monitor the process over time to detect shifts and trends in data in real time.^18^ Furthermore, the run chart shows sustainability of this QI project throughout the pandemic despite the unprecedented challenges that may have arisen during this time and clearance of 52 patients on the waiting list prior to biopsies starting while maintaining efficient waiting times. This waiting list was cleared within 12 weeks while receiving new referrals.

It is possible that due to the unprecedented nature of this pandemic, there were less patients attending their primary healthcare provider for routine PSA tests and subsequently less target referrals being made to the service.^19^ This in combination with reduced clinical and surgical activity means that resources could be focussed in starting this new service, which may have provided time for the service to quickly implement this new service and work out any early issues that may have arisen. Furthermore, the introduction of mpMRI as a first‐line investigation prior to any TP biopsy may mean less patients would be subjected to TP biopsy than those who would have had TRUS biopsy as a fist line.

Other additional benefits included the following:

1. Less patients' histology being discussed at MDT, streamlining the MDT,
2. No document cases trust wide of admissions for post‐biopsy sepsis.
3. Use of antibiotics used in this procedure was reduced to less than 3% of cases.
4. Two hundred eighty additional GA operations were achieved due to extra theatre and staff capacity.

The results have shown that there was a statistically significant improvement in the number of days waiting for a TP biopsy pre‐lockdown when compared with lockdown (*U* = 55.5, *p* ≤ 0.001). The mean waiting time was reduced from 145 to 23 days post‐introduction of the gateway pathway; however, this could be explained by long wait active surveillance cases. Interestingly, when observing the significant increase in clinical activity between 1 June 2021 and 29 November 2021, we can see that despite the increase in clinical activity in trust when comparing 12 May 2020–5 November 20 (*n* = 144) to 1 June 2021–29 November 2021 (*n* = 271), there was no statistically significant increase in the mean waiting time for a biopsy (23 days during lockdown, 30 days upon easing of restrictions [*U* = 18461.5 *p* = 0.36812]). This shows that a nurse‐led TP biopsy service under LA is a resilient service able to withstand pressures of increased clinical activity and the transition to BAU. There is current an ongoing drive within the trust to train a second ANP to perform TP biopsy, which may lead to a further efficiency and durability and possible further reductions in patient wait times, which has been identified as a further limitation and an area for further improvement.

This study may be of interest to a number of trusts in the UK as converting a TP biopsy service. Previous limitations from a large cohort study and cost analysis by Roberts et al. have suggested that there are significant savings when transferring from a TRUS to a consultant led, GA, TP approach.^20^ By progressing to a nurse‐led LA, TP approach further resources such as theatres, anaesthetists, operating department theatres, scrub nurses and urologist may be freed up saving the trust money in the long term while ensuring ANP's can use their specialist skills to their full potential, perhaps leading to greater job satisfaction in the long term. Consultant‐led GA TP biopsies could instead be utilised as a second‐line investigation for those requiring re‐biopsy or in select cases. Further randomised control trials would be useful to determine parity in accuracy between LA TP biopsies and GA TP biopsies.

## 5 CONCLUSIONS

In conclusion, it is clear that regardless of the pandemic urology departments nationally should continue their efforts towards an LA nurse‐led TP biopsy service. The implementation of this QI project shows that a nurse‐led LA TP biopsy service is not only something achievable but also sustainable regardless of the disruption caused by the current international circumstances. By centralising the biopsy service, we have improved business performance across the trust, and sessions that were often time consuming were freed up across three other sites, releasing consultant urology staff (five consultant sessions, that is, over half of a consultant post) to undertake more complex duties and release on average >1 sessions a week of theatre time. It has eliminated post‐biopsy sepsis and as a result saved inpatient beds in our trust as well as high‐dependency care unit/intensive care unit beds.

Not only may this service maximise use of resources available to urology departments but it may make further sense economically in the long term. The reduction in waiting lists and efficiency of our service has shown that nothing can stop a well‐planned and well‐managed quality improvement project while the team behind it remain determined to improve patient outcomes.

## AUTHOR CONTRIBUTIONS

David Winters is the lead author who wrote paper, collected data and performed the analysis. Ashley Mehmi collected data. Amar Odedera collected data and aided in article writing. Lydia Wilson collected data. Joey Ancheta performed the biopsies and implemented the service. Sally Buttleman performed the biopsy and implemented the service. Paula Allchorne implemented the service. Prabhakar Rahman implemented service. Shahid Khan is one of the study leads, who helped in the project implementation and data capture. James S. A. Green is one of the study leads, who helped develop the original idea and QI study design.

## DECLARATION OF CONFLICT OF INTEREST

None.
